# Implications of Regionalizing Care in the Developing World: Impact of Distance to Referral Center on Compliance to Biopsy Recommendations in a Brazilian Prostate Cancer Screening Cohort

**DOI:** 10.1155/2021/6614838

**Published:** 2021-06-22

**Authors:** Alexis R. Freedland, Roberto L. Muller, Cathrine Hoyo, Elizabeth L. Turner, Patricia G. Moorman, Eliney F. Faria, Gustavo F. Carvalhal, Rodolfo B. Reis, Edmundo C. Mauad, Andre L. Carvalho, Stephen J. Freedland

**Affiliations:** ^1^Department of Epidemiology, UCI School of Medicine, University of California, Irvine, CA, USA; ^2^Division of Urology, Center of Oncologic Research, Florianopolis, Santa Catarina, Brazil; ^3^Department of Biological Sciences, North Carolina State University, Raleigh, NC, USA; ^4^Global Health Institute, Duke University, Durham, NC, USA; ^5^Department of Biostatistics and Bioinformatics, Duke University Graduate School, Durham, NC, USA; ^6^Department of Community and Family Medicine, Cancer Control and Population Sciences, Duke Cancer Institute, Durham, NC, USA; ^7^Division of Urologic Oncology and Laparoscopy, Barretos Cancer Hospital, Barretos, São Paulo, Brazil; ^8^Research Support Center, Barretos Cancer Hospital, Barretos, São Paulo, Brazil; ^9^Division of Urology, Ribeirao Preto Medical School of Sao Paulo University (USP), Ribeirao Preto, São Paulo, Brazil; ^10^Department of Preventative Medicine, Barretos Cancer Hospital and Pio XII Foundation, Barretos, São Paulo, Brazil; ^11^Division of Urology, Department of Surgery, Cedars-Sinai Medical Center, Los Angeles, CA, USA

## Abstract

Given growing specialization in medical care, optimal care may require regionalization, which may create access barriers. We tested this within a large prostate cancer (PC) screening program in Brazil. In 2004–2007, Barretos Cancer Hospital prospectively screened men for PC throughout rural Brazil. Men with abnormal screen were referred for follow-up and possible biopsy. We tested the link between distance from screening site to Barretos Cancer Hospital and risk of noncompliance with showing up for biopsy, PC on biopsy and, among those with PC, PC grade using crude and multivariable logistic regression analysis. Among 10,467 men undergoing initial screen, median distance was 257 km (IQR: 135–718 km). On crude and multivariable analyses, farther distance was significantly linked with biopsy noncompliance (OR/100 km: 0.83, *P* < 0.001). Among men who lived within 150 km of Barretos Cancer Hospital, distance was unrelated to compliance (OR/100 km: 1.09, *P*=0.87). There was no association between distance and PC risk or PC grade (all *P* > 0.25). In Brazil, where distances to referral centers can be large, greater distance was related to reduced biopsy compliance in a PC screening cohort. Among men who lived within 150 km, distance was unrelated to compliance. Care regionalization may reduce access when distances are large.

## 1. Introduction

A 2011 study of overall cancer mortality in Brazilian state capitals (urban areas) vs. outlying municipalities (rural areas) from 1980 to 2006 found a mortality increase of more than 100% for all cancer types [1]. While prostate cancer (PC) mortality increased over time in all regions, mortality increased faster among men in rural areas. Although the exact reasons for this increase are unclear, it is conceivable that rural men have reduced access to healthcare and thus do not receive the same benefits of early detection and treatment.

Given the growing number of aging people with varied health needs and growing specialization in medical care [2–4], there is increasing realization that optimal care for certain procedures may require regionalization [5, 6]. While this comes with the benefit of creating high-volume centers to drive optimal outcomes, this also comes at the cost of increased burden to travel, including obtaining transportation [7], loss of wages, and being away from home [6, 8]. A 2016 multinational systematic review of 108 studies from developed countries evaluated the association between distance to healthcare services and subject outcomes and found some studies showed greater distances negatively impacted access to care [9]. Importantly, the distances were typically ≤100 miles. Given the large distances needed to travel for care in Brazil, there is concern that subjects may not follow up for required care [10]. It is likely the distance subjects are willing to travel depends on the perceived benefits of receiving such specialized care. As such, the nature of the medical condition and the distance to the care facility may dictate whether the benefits of regionalization outweigh the barriers this creates for access.

The 2004–2007 Barretos Cancer Hospital (BCH) medical Mobile Cancer Prevention Screening Unit (MCPSU) is the largest PC screening study in South America [11]. Due to its study design, wherein men were initially screened in local communities and those with abnormal screens were referred to a care facility often hundreds of kilometers (km) away, the MCPSU provides a unique opportunity to study the impact of care regionalization on PC in Brazil. We hypothesized greater distance will have a negative impact on receipt of follow-up care after PC screening and only those with the highest PC risk will adhere due to greater perceived benefits of care, leading to a positive link between distance and aggressive PC among those who adhere.

## 2. Materials and Methods

### 2.1. Data Collection

After obtaining institutional review board approval, we conducted a cross-sectional study of 11,117 men who underwent initial PC screening as a part of the MCPSU from 2004 to 2007 in 231 cities in Southern Brazil. Because these areas had limited access to health services, PC screening took place on BCH MCPSUs. Screening was targeted to specific low socioeconomic status (SES) cities. Distances between local screening sites and regional diagnostic and treatment facilities at BCH were estimated using the shortest route obtained between each participating city and the address of BCH calculated by Google Maps (Google LLC, Mountain View, CA, USA).

Enrollment methods were previously described [11, 12] and informed consent was obtained from each participant. Participants received free PC screens from MCPSU personnel using on-site prostate specific antigen (PSA) testing and digital rectal exams (DRE). The same staff performed PSAs and DREs at every site. Men with total PSA (tPSA) > 4.0 ng/mL, tPSA 2.5–4.0 ng/mL with percent-free PSA (pfPSA) ≤15%, or DRE suspicious of PC were contacted by mail or phone and referred to BCH for follow-up for possible biopsy. Men were required to follow up within six months after screen. Once at BCH, repeat PSA and DRE were performed. Men whose indications were confirmed were recommended to undergo biopsy.

### 2.2. Study Participants

**Figure 1:**
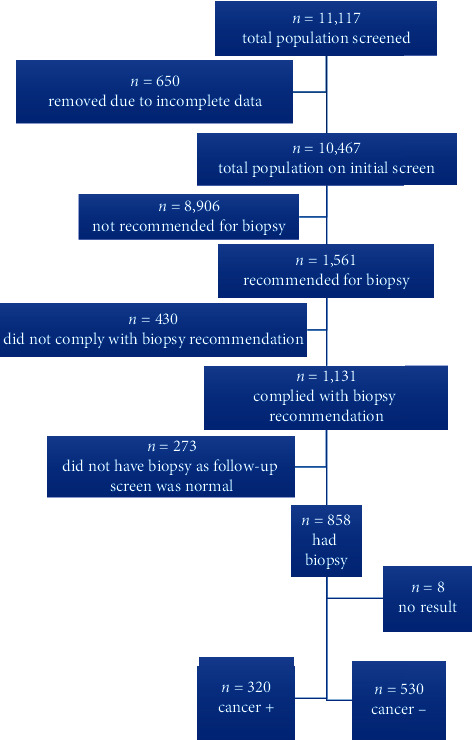
Consort flow diagram describing the progression of subjects through the study.

Among 11,117 men undergoing initial screen, we excluded men missing data on education (*n* = 6) or PSA (*n* = 644), leaving 10,467 (94%) men. Of these, 1,561 (15%) were referred for further evaluation, of which 1,131 (72%) complied and 430 (28%) did not. Of the 1,131 men who presented to BCH, all underwent a confirmation screening. 273 had normal screenings and were no longer recommended for biopsy and eight were excluded from analysis due to inconclusive biopsy pathology. Therefore, for secondary analyses, we examined 850 men for PC on biopsy and PC grade (Figure 1).

### 2.3. Statistical Analysis

Associations between clinical or demographic factors for compliant and noncompliant men were evaluated using a two-sample *t*-test with equal variances for normally distributed continuous variables, Wilcoxon rank-sum test for nonnormally distributed continuously variables, and Pearson's chi-square test for categorical variables. In multivariable analyses, nonnormally distributed variables were logarithmically transformed to obtain normal distribution of the data. Variables ascertained via questionnaire included age (continuous, years), initial screening tPSA (continuous, logarithmically transformed), distance from screening site to BCH (categorical, <250, 250–500, 500–1000, ≥1000 km, and logarithmically transformed continuous), education level (categorical, illiterate, incomplete primary, complete primary, high school, and college), PC family history (yes/no), family history of other cancers (yes/no), year of screening (categorical, 2004, 2005, 2006, and 2007), and DRE findings (normal/abnormal). Primary analyses used distance as a continuous logarithmically transformed variable. To provide clinical context, we also examined distance categorically in 250 km categories. Logistic regression was used to test the association between distance and biopsy noncompliance. Multivariable models were adjusted for the aforementioned covariates. We used a locally weighted smoothed scatterplot (LOWESS) to graphically depict the association between distance and biopsy compliance. Based upon visual review of the LOWESS, a post hoc subset analysis was conducted testing the association between distance and compliance among men with shorter distances (150 km cutoff).

Secondary analyses evaluated the association between distance and PC risk on biopsy using crude and multivariable models, adjusting for the aforementioned covariates. Finally, we used multinomial logistic regression to assess the association between distance and PC grade defined as no PC (referent group), low-grade PC (Grade Group 1), and high-grade PC (Grade Group ≥2). [13] All statistical analyses were performed using Stata 13.1 (StataCorp, College Station, TX, USA). Two-tailed *P* values of ≤0.05 were considered statistically significant.

## 3. Results

### 3.1. Baseline Clinical Characteristics and Cancer Details among Compliant Men

Noncompliant men (*n* = 430, 27.5%) were older at screen (mean: 68 vs. 66 years, *P* < 0.001), had higher enrollment tPSA (4.9 vs. 4.2 ng/mL, *P* < 0.001), and were less likely to have an abnormal DRE (19.5% vs. 33.4%, *P* < 0.001) than compliant men (*n* = 1,131, 72.5%). Noncompliant men had less education (illiterate + incomplete primary school: 91.7% vs. 89.2%, *P* < 0.001) and were more likely to live farther away from BCH (median: 921 vs. 225 km, *P* < 0.001). There were no statistically significant differences in family history for any cancer (*P*=0.07) or PC (*P*=0.15) by compliance status (Table 1).

Of the 850 compliant men who underwent a biopsy, 320 (37.7%) had PC, of which 65.1% had low-grade PC (Grade Group 1) and most had Stage 1 disease (74.5%). The median PSA of men on biopsy was 7 ng/mL (IQR: 4–16 ng/mL) and the median number of positive cores was 3/12 (IQR: 2–5, Table 1).

### 3.2. Distance and Noncompliance

On crude analysis, noncompliant men lived further from BCH (*P* < 0.001). Specifically, median (IQR) distance for compliant men was 225 km (119–414 km) vs. 921 km (333–1603 km) for noncompliant men. To put this in a more clinically useful form, we also evaluated distance in 250 km categories and noted that, relative to men who lived <250 km from BCH, those who lived farther away were more likely to be noncompliant with follow-up recommendations (250–500 km: OR: 2.00, 95% CI: 1.40–2.85; 500–1000 km: OR: 5.88, 95% CI: 4.07–8.51; ≥1000 km: OR: 15.98, 95% CI: 11.41–22.38, *P* < 0.001; Table 2). After adjusting for clinical and demographic factors, results were largely unchanged (Table 2).

**Figure 2:**
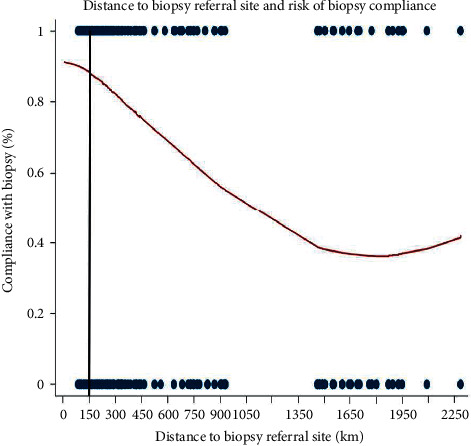
LOWESS smoothened curve evaluating the univariable association between distance to biopsy referral site and compliance.

Figure 2 shows the relationship between distance and compliance. Compliance declines linearly with distance until distance reaches ∼1,500 km, after which numbers are small, and the beginning of the association had a less steep decline. To explore the earlier part of the curve, we chose a distance of 150 km (chosen post hoc) and examined, within this limited range, whether distance impacted compliance. On multivariable analysis with distance as a continuous variable, distance was unrelated to compliance when examining men who lived within 150 km of BCH (OR/100 km: 1.09, 95% CI: 0.40–2.91, *P*=0.87).

### 3.3. Distance and Biopsy Outcomes

On crude analysis, there was no statistically significant association between distance and PC on biopsy (*P*=0.45) as well as both low-grade PC (*P*=0.56) and high-grade PC (*P*=0.50, Table 3). Results were unchanged on multivariable analysis for overall PC risk (*P*=0.80) and low (*P*=0.79) and high-grade disease (*P*=0.94).

## 4. Discussion

There is increased interest in understanding the impact of regionalizing care on access. The impact of greater distance on receipt of follow-up care after PC screening in a developing country like Brazil, where large proportions of the population reside in rural areas, is unknown. We tested if there was a link between distance and compliance to follow-up recommendations or being diagnosed with aggressive PC among men who underwent initial PC screening in their local community. We found that longer distance was significantly associated with follow-up noncompliance (OR/100 km: 0.83, *P* < 0.001). However, among men who lived within 150 km of the referral center, distance was unrelated to compliance (OR/100 km: 1.09, *P*=0.87). We found no association between distance and PC risk or PC grade on biopsy (all *P* > 0.25). Our results show that in Brazil, where distances can be very large, longer distance can negatively impact follow-up among men undergoing PC screening manifesting as a barrier to care. Whether similar associations are seen in other countries and healthcare systems requires further study.

While distance to care is a potential barrier restricting access, data evaluating the association between distance and compliance with cancer screening are limited. Though our study is the first to analyze the association between distance and PC in the developing world, few studies addressed this issue in the US [4, 14–16]. One single-center study examined the association between distance and missed clinic appointments among men suspected of or being treated for PC [14]. Among 1,341 scheduled clinic encounters for 576 patients, greater distance was associated with *improved* compliance (compliant median = 11.8 mi vs. missed appointment = 10.4 mi, *P*=0.04). Investigators found that greater distance was not a significant barrier to access to healthcare, though the relatively small distances traveled in this study may not be applicable in developing countries where distances required to travel are often significantly larger [4, 15]. Indeed, our study agrees that small distances do not create barriers for access. Our findings are in line with a study from the Prostate, Lung, Colon, and Ovarian Cancer Screening Trial, which found that subjects who lived farthest away (units unknown) were more likely to be noncompliant with adherence to first-round screening than subjects who lived closer (near: 14%; midrange: 18%; far: 20%). However, the association between farther distance and poor compliance became nonsignificant on multivariable adjustment for SES, lifestyle, and demographic confounders [16]. Unfortunately, in our study, we were not able to adjust for SES defined by wealth and occupation or other lifestyle factors as these data were unavailable. Collectively, while these data suggest that large distances may be linked with poor compliance, this had never been studied in a developing country like Brazil.

To explore the barriers that regionalization can create, we tested the link between distance and risk of noncompliance with showing up to have follow-up and a possible biopsy in a population-based PC screening cohort in Brazil. We found that increased distance to referral facility was significantly associated with increased risk of noncompliance to biopsy recommendations (2–16 times more likely depending on distance). Importantly, when distances were <150 km, distance was unrelated to compliance. We found no association between distance and PC detection or grade on biopsy. These data suggest that while regionalization of care may *in theory* improve quality, it comes at the cost of reduced compliance and thus reduced access. This represents a significant barrier to optimal care if distances are large. Regarding PC screening and biopsy, our data suggest distances up to 150 km do not create barriers for care in Brazil. Alternative thresholds may apply for other services and in other countries.

While distance created a barrier for compliance, we hypothesized that men who stood the most to benefit from screening (i.e., those at higher risk of aggressive PC) would be more compliant creating a positive association between greater distance and more aggressive tumors. Indeed, such an association was found in breast cancer where increased distance (highest category ≥58 km) to screening site was associated with higher stage at diagnosis (*P*=0.037) [17]. Whether this suggests that people who live further distances only present for screening if they have higher risk disease, or whether distance-related barriers leading to delayed diagnosis and advanced disease is unknown, we did not find a relationship consistent with either hypothesis. However, it is worth noting that, among compliant men, the upper IQR for tPSAs = 7.1 ng/mL, a level that is rarely associated with symptoms from advanced PC. As such, the majority of men in our study would have been asymptomatic or have minimal, less aggressive disease, though data on symptoms was unavailable. Further, men who were recommended for follow-up were not informed of their actual PSA values, so they may not have been aware of their actual PC risk. Instead, they were told that they were at “increased” risk. It is unknown whether our results would have been different if men were informed of their PC risk via receipt of actual PSA values or using a risk calculator to estimate PC risk. Indeed, others have also found distance was related to stage at diagnosis, albeit in colorectal and lung cancer [18–21]. Thus, though we found no link between distance and grade, given conflicting literature from other cancers, more research is needed on this topic. Of note, only ∼37% of men in our study had PC on initial biopsy, 65% of which were Grade Group 1 PCs. Viewed alternatively, only 13% of men in our study had higher grade disease (Grade Group 2–5). Whether our results would have differed in a cohort that included a higher percent of high-risk men is unknown.

Our study has limitations. First, these data were not collected with research intent. However, as any misclassifications in the data tend to bias results to the null, it is possible that the association between distance and poor compliance may be stronger than observed. Additional information such as SES, race, body composition, and comorbidities was unavailable. How much these would have influenced our results is unknown. Additional studies are needed to understand the impact of other factors on compliance and its effect on long-term PC outcomes. The outcome of noncompliant men is unknown. Perhaps they received appropriate care elsewhere or perhaps noncompliance delayed diagnosis of their PC and led to worse outcomes. Measurements of distance are subjective: we chose distance traveled by road, though other evaluations of distance may have alternative findings. The impact of distance on received care would be influenced not only by physical distance but the infrastructure available to overcome the barriers distance presents (e.g., quality of roads, airports, etc.). Therefore, further analyses are needed to evaluate how transportation presents as barrier. It is possible that wealth may modify the association between distance and compliance. Specifically, more affluent people may be less sensitive to distance as a barrier in that they can afford to travel easily. Unfortunately, as individual level income data were not available, we could not address this possibility. Finally, while our study assessed the negative impact of regionalization, due to the limits of the data, we are unable to assess potential benefits of regionalization (i.e., improved quality of care) or examine survival or cost-benefit analyses. Further studies are needed to examine the net benefits vs. risks of regionalization, particularly for other diseases and countries.

Despite these limitations, our study has several key strengths. Given the high level of illiteracy, investigators contacted men whose biopsy indications were suspicious of PC by mail and by phone. Including a phone call as a notification method ensured that the men received the information and were able to understand it. If notifications were only by mail, this could be a major explanatory variable for the low follow-up rates in illiterate men. Secondly, contamination issues commonly seen in large-scale screening studies were minimized as we restricted analyses to first screens. Thirdly, there were standardized methods of data collection for each enrolled subject with over 94% of men having complete data. The fact that all data were collected prior to patients knowing their screening results minimized recall bias. The same personnel performed DRE exams throughout the study period. This ensured uniformity in classification of PC risk. Regarding postscreen biopsies, there was limited variability in the number of biopsy cores taken (median: 12, IQR: 10–14), consistent with global standards to optimize PC detection [22]. Most notably, when screening programs are instituted, it is important to ensure that there are provisions for people with positive screens to be able to receive appropriate follow-up and care. With such plans in place, our study is comprised of data from a large cohort of rural Brazilian men, who have previously not been well-studied, providing unique insights into an understudied population.

## 5. Conclusion

Among Brazilian men undergoing initial PC screening, we found greater distance from local screening site to regional follow-up care facility was a significant risk factor for noncompliance to biopsy recommendations. However, when distances were <150 km, distance was unrelated to biopsy compliance. Finally, we found no association between distance and risk of PC or PC grade on biopsy among those who underwent a biopsy. While regionalization of care may in theory improve quality, it comes at the cost of reduced compliance and reduced access and represents a significant barrier to optimal care if distances are large.

## Figures and Tables

**Table 1:** Baseline characteristics of men undergoing initial PC screening by Brazilian mobile medical units stratified by compliance to biopsy recommendation (*n* = 1,561) and PC details among compliant men who underwent biopsy (*n* = 318).

Baseline characteristics	Compliant men	Noncompliant men	*P* ^+^
	No. (%)		
	*N* = 1,131 (72.5)	*N* = 430 (27.5)	
*Educational level attained*			
Illiterate	279 (24.7)	165 (38.4)	<0.001
Incomplete primary	729 (64.5)	229 (53.3)	
Complete primary	86 (7.6)	17 (4.0)	
High school	30 (2.7)	15 (3.5)	
College	7 (0.6)	4 (0.9)	

*Year of screening*			
2004	230 (20.3)	82 (19.1)	<0.001
2005	355 (31.4)	80 (18.6)	
2006	333 (29.4)	102 (23.7)	
2007	213 (18.8)	166 (38.6)	

*Positive family history*			
PC	56 (5.0)	14 (3.3)	0.15
Any cancer	122 (10.8)	33 (7.7)	0.07
DRE suspicious of PC	378 (33.4)	84 (19.5)	<0.001

*Cancer status*			
Positive	320 (37.7)	—	—
Negative	530 (62.4)	—	—

*Grade group*			
I (Gleason 2–6)	207 (65.1)	—	—
II-III (Gleason 7)	85 (26.7)	—	—
IV-V (Gleason 8–10)	26 (8.2)	—	—

*Clinical stage*			
Stage I	237 (74.5)	—	—
Stage II	42 (13.2)	—	—
Stage III	16 (5.0)	—	—
Stage IV	23 (7.2)	—	—

	Mean (SD)		*P* ^*∗*^
Age (years)	66 (9.0)	68 (9.3)	<0.001

	Median (IQR)		*P* ^#^
tPSA (ng/mL)	4.2 (2.6–7.1)	4.9 (3.5–7.7)	<0.001
Distance from Barretos (km)	225 (119–414)	921 (333–1603)	<0.001
Total number of positive cores^@^	3 (2–5)	—	—
Total number of cores taken^@^	12 (10–14)	—	—
Prostate volume (cc)^@^	33 (25–46)	—	—
tPSA (ng/mL)^@^	7 (4–16)	—	—

Statistical analyses: ^*∗*^*t*-test; ^+^chi-squared test; ^#^rank-sum. DRE: digital rectal exam; IQR: interquartile range; km: kilometer; *P*: *P* value; PC: prostate cancer; tPSA: total serum prostate specific antigen (ng/mL). ^@^Among 318 men with available data.

**Table 2:** Association between distance and risk of noncompliance.

Model: logistic regression	Distance variable format	Distance (km)	OR	95% CI	*P* value
Unadjusted	Continuous	—	2.81	2.48–3.19	<0.001
	Categorical relative to 0–249 km	250–499	2.00	1.40–2.85	<0.001
		500–999	5.88	4.07–8.51	
		≥1000	15.98	11.41–22.38	

Multivariable	Continuous	—	2.71	2.37–3.09	<0.001
	Categorical relative to 0–249 km	250–499	1.87	1.30–2.70	<0.001
		500–999	5.36	3.65–7.87	
		≥1000	14.91	10.45–21.28	

Multivariable analysis adjusted for age, log PSA, education, family history of cancer, family history of PC, DRE, and screening year. CI: confidence interval; km: kilometers; OR: odds ratio.

**Table 3:** The association between distance and risk of PC and PC grade (*n* = 850).

Association between distance and risk of PC					
Model: logistic regression	Distance variable format	Distance (km)	OR	95% CI	*P* value
Unadjusted	Continuous	—	1.05	0.92–1.20	0.45
	Categorical relative to 0–249 km	250–499	1.05	0.76–1.46	0.37
		500–999	0.87	0.54–1.40	
		≥1000	1.45	0.91–2.30	

Multivariable	Continuous	—	0.98	0.85–1.14	0.80
	Categorical relative to 0–249 km	250–499	0.96	0.67–1.37	0.39
		500–999	0.69	0.41–1.17	
		≥1000	1.22	0.74–2.03	

Association between distance and PC grade (relative to no PC)					
Model: multinomial logistic regression	Distance variable format	Distance (km)	OR	95% CI	*P* value

*Cancer outcome*: *low-grade PC (Grade Group I)*					
Unadjusted	Continuous	—	1.04	0.91–1.20	0.56
	Categorical relative to 0–249 km	250–499	0.97	0.69–1.36	0.27
		500–999	0.86	0.53–1.40	
		≥1000	1.38	0.86–2.22	
Multivariable	Continuous	—	0.98	0.84–1.14	0.79
	Categorical relative to 0–249 km	250–499	0.90	0.62–1.30	0.32
		500–999	0.69	0.40–1.17	
		≥1000	1.20	0.72–2.00	

*Cancer outcome*: *high-grade PC* (*Grade Group ≥*2)					
Unadjusted	Continuous	—	1.13	0.79–1.64	0.50
	Categorical relative to 0–249 km	250–499	2.44	1.03–5.79	0.27
		500–999	0.96	0.20–4.48	
		≥1000	2.39	0.72–7.93	
Multivariable	Continuous	—	0.98	0.65–1.49	0.94
	Categorical relative to 0–249 km	250–499	2.15	0.83–5.57	0.32
		500–999	0.63	0.12–3.33	
		≥1000	1.47	0.34–6.41	

Multivariable analysis adjusted for age, log PSA, education, family history of cancer, family history of PC, DRE, and screening year. CI: confidence interval; km: kilometers; OR: odds ratio; PC: prostate cancer; RRR: relative risk ratio.

## Data Availability

The data used to support the findings of this study are restricted by the IRB of Barretos Cancer Hospital in order to protect patient privacy.

## Abbreviations

BCH: Barretos Cancer Hospital
LOWESS: Locally weighted smoothed scatterplot
MCPSU: Mobile Cancer Prevention Screening Unit.
